# Generalization of the Partitioning of Shannon Diversity

**DOI:** 10.1371/journal.pone.0090289

**Published:** 2014-03-06

**Authors:** Eric Marcon, Ivan Scotti, Bruno Hérault, Vivien Rossi, Gabriel Lang

**Affiliations:** 1 AgroParisTech, UMR Écologie des Forêts de Guyane, Kourou Cedex, France; 2 INRA, UMR Écologie des Forêts de Guyane, Kourou Cedex, France; 3 CIRAD, UMR Écologie des Forêts de Guyane, Kourou Cedex, France; 4 AgroParisTech, UMR 518 Math. Info. Appli., Paris, France; 5 INRA, UMR 518 Math. Info. Appli., Paris, France; CNRS - Université Lyon 1, France

## Abstract

Traditional measures of diversity, namely the number of species as well as Simpson's and Shannon's indices, are particular cases of Tsallis entropy. Entropy decomposition, *i.e.* decomposing gamma entropy into alpha and beta components, has been previously derived in the literature. We propose a generalization of the additive decomposition of Shannon entropy applied to Tsallis entropy. We obtain a self-contained definition of beta entropy as the information gain brought by the knowledge of each community composition. We propose a correction of the estimation bias allowing to estimate alpha, beta and gamma entropy from the data and eventually convert them into true diversity. We advocate additive decomposition in complement of multiplicative partitioning to allow robust estimation of biodiversity.

## Introduction

Diversity partitioning means that, in a given area, the gamma diversity 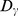 of all individuals found may be split into within (alpha diversity, 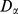) and between (beta diversity, 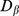) local assemblages. Alpha diversity reflects the diversity of individuals in local assemblages whereas beta diversity reflects the diversity of the local assemblages. The latter, 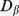, is commonly derived from 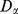 and 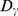 estimates [1]. Recently, a prolific literature has emerged on the problem of diversity partitioning, because it addresses the issue of quantifying biodiversity at large scale. Jost's push [2]–[5] has helped to clarify the concepts behind diversity partitioning but mutually exclusive viewpoints have been supported, in particular in a forum organized by Ellison [6] in *Ecology*. A recent synthesis by Chao *et al.* [7] wraps up the debate and attempts to reach a consensus. Traditional measures of diversity, namely the number of species as well as Simpson's and Shannon's indices, are all special cases of the Tsallis entropy [8], [9]. The additive decomposition [10] of these diversity measures does not provide independent components but Jost [3] derived a non-additive partitioning of entropy which does.

A rigorous vocabulary is necessary to avoid confusion. *Unrelated* or *independent* (sensu [7]) means that the range of values of 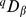 is not constrained by the value of 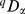, which is a desirable property. *Unrelated* is more pertinent than *independent* since diversity is not a random variable here, but *independent* is widely used, by [3] for example. We will write *independent* throughout the paper for convenience. We will write *partitioning* only when independent components are obtained and *decomposition* in other cases.

Tsallis entropy can be easily transformed into Hill numbers [11]. Jost [3] called Hill numbers *true diversity* because they are homogeneous to a number of species and have a variety of desirable properties that will be recalled below. We will call *diversity* true diversity only, and *entropy* Simpson and Shannon indices as well as Tsallis entropy. The multiplicative partitioning of true 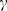 diversity allows obtaining independent values of 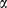 and 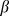 diversity when local assemblages are equally weighted.

However, we believe that the additive decomposition of entropy still has something to tell us. In this paper, we bring out an appropriate mathematical framework that allows us to write Tsallis entropy decomposition. We show its mathematical equivalence to the multiplicative partition of diversity. This is simply a generalization of the special case of Shannon diversity [12]. Doing so, we establish a self-contained (*i.e.* it does not rely on the definitions of 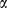 and 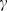 entropies) definition of 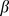 entropy, showing it is a generalized Jensen-Shannon divergence, *i.e* the average generalized Kullback-Leibler divergence [13] between local assemblages and their average distribution. Beyond clarifying and making explicit some concepts, we acknowledge that this decomposition framework largely benefits from a consistent literature in statistical physics. In particular, we rely on it to propose bias corrections that can be applied to Tsallis entropy in general. After bias correction, conversion of entropy into true diversity provides independent, easy-to-interpret components of diversity. Our findings complete the well-established non-additive (also called pseudo-additive) partitioning of Tsallis entropy. We detail their differences all along the paper.

## Methods

Consider a meta-community partitioned into several local communities (let 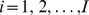 denote them). 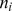 individuals are sampled in community 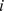. Let 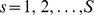 denote the species that compose the meta-community, 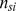 the number of individuals of species 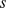 sampled in the local community 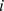, 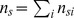 the total number of individuals of species 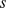, 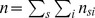 the total number of sampled individuals. Within each community 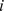, the probability 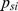 for an individual to belong to species 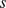 is estimated by 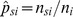. The same probability for the meta-community is 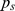. Communities may have a weight,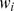, satisfying 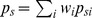. The commonly-used 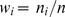 is a possible weight, but the weighting may be arbitrary (*e.g.* the sampled areas).

We now define precisely entropy. Given a probability distribution 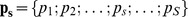, we choose an information function 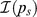, which is a decreasing function of 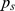 having the property 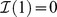: information is much lower when a frequent species is found. Entropy is defined as the average amount of information obtained when an individual is sampled [14]:
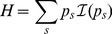
(1)


The best-known information function is 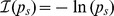. This defines the entropy of Shannon [15]. 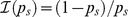 yields the number of species minus 1 and 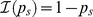, Simpson's [16] index. Relative entropy is defined when the information function quantifies how different an observed distribution 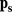 is different from the expected distribution 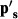. The Kullback-Leibler [17] divergence is the best-known relative entropy, equal to 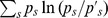. Shannon's beta entropy has been shown to be the weighted sum of the Kullback-Leibler divergence of local communities, where the expected probability distribution of species in each local community is that of the meta-community [12], [18]:
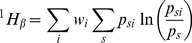
(2)


Let us define 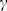 as the meta-community's diversity, 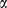 as local communities' diversities, and 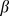 as diversity between local communities. Tsallis 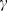 entropy of order 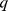 is defined as:
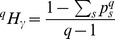
(3)and the corresponding 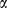 entropy in the local community 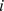 is:
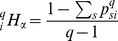
(4)


The natural definition of the total 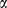 entropy is the weighted average of local community's entropies, following Routledge [19]:
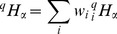
(5)


This is the key difference between our decomposition framework and the non-additive one. Jost [3] proposed another definition, 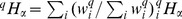, *i.e.* the normalized *q*-expectation of the entropy of communities [20] rather than their weighted mean. It is actually a derived result, see the discussion below. Our results rely on Routledge's definition (see Appendix S1).


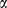
 and 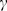 diversity values are given by Hill numbers 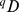, called “numbers equivalent” or “effective number of species”, *i.e.* the number of equally-frequent species that would give the same level of diversity as the data [14]:
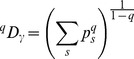
(6)


Routledge 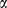 diversity is:
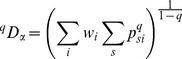
(7)


Combining (3) and (6) yields:
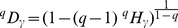
(8)


We also use the formalism of deformed logarithms, proposed by Tsallis [21] to simplify manipulations of entropy. The deformed logarithm of order 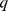 is defined as:
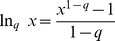
(9)


It converges to 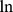 when 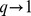.

The inverse function of 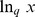 is the deformed exponential:
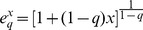
(10)


The basic properties of deformed logarithms are:
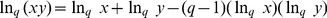
(11)

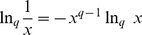
(12)

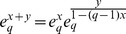
(13)


Tsallis entropy can be rewritten as:
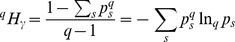
(14)


Diversity and Tsallis entropy are transformations of each other:
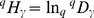
(15)

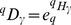
(16)


### Decomposing diversity of order 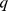


We start from the multiplicative partitioning of true diversity.
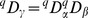
(17)


If community weights are equal, 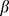 diversity is independent of 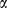 diversity (it is whatever the weights if 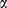 diversity is weighted according to Jost, but this is not our choice). We will consider the unequal weight case later.


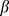
 diversity is the equivalent number of communities, *i.e.* the number of equally-weighted, non-overlapping communities that would have the same diversity as the observed ones.

We want to explore the properties of entropy decomposition. We calculate the deformed logarithm of equation (17):
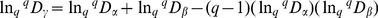
(18)

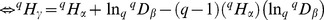
(19)


Equation (19) is Jost's partitioning framework (equation 8f in [3]). Jost retains 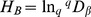 as the 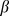 component of entropy partitioning. It is independent of 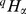 (they are respective transformations of independent 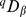 and 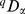), contrarily to the 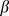 component of the additive decomposition [10], [22] defined as 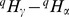


After some algebra requiring Routledge's defintiion of 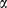 diverity detailed in Appendix S1, we obtain from equation (19):
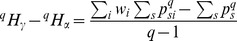
(20)


The right term of equation (20) is a possible definition of the 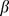 component of additive decomposition. It can be much improved if we consider 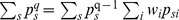 and rearrange equation (20) to obtain:
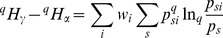
(21)


We obtained the 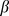 entropy of order 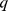. It is the weighted average of the generalized Kullback-Leibler divergence of order 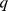 (previously derived by Borland *et al.* [13] in thermostatistics) between each community and the meta-community:
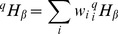
(22)

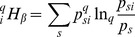
(23)

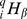
 converges to the Kullback-Leibler divergence when 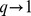.

The average Kullback-Leibler divergence between several distributions and their mean is called Jensen-Shannon divergence [23], so our 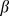 entropy 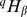 can be called *generalized Jensen-Shannon divergence*. It is different from the non-logarithmic Jensen-Shannon divergence [24] which measures the difference between the equivalent of our 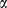 entropy and 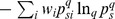 (the latter is not Tsallis 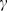 entropy).

Our results are summarized in Table 1, including transformation of entropy into diversity. The partition of entropy of order 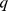 is formally similar to that of Shannon entropy. It is in line with Patil and Taillie's [14] conclusions: 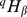 is the information gain attributable to the knowledge that individuals belong to a particular community, beyond belonging to the meta-community.

**Table 1:** Values of entropy and diversity for generalized entropy of order 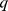 and Shannon entropy.

Diversity measure	Generalized entropy	Shannon
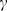 entropy	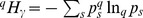	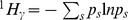
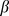 entropy	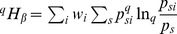	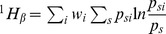
True 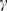 diversity (Hill number)	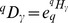	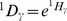
True 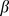 diversity (numbers equivalent)	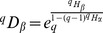	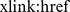

The deformed logarithm formalism allows presenting all orders of entropy as a generalization of Shannon entropy. Generalized 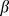 entropy is a generalized Kullback-Leibler divergence, *i.e.* the information gain obtained by the knowledge of each community's composition beyond that of the meta-community. Robust estimation of the entropy of real communities requires estimation bias correction introduced in the text.

### Information content of generalized entropy

Both 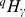 and 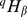 must be rearranged to reveal their information function and explicitly write them as entropies. Straightforward algebra yields:
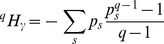
(24)

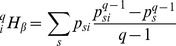
(25)


The information functions respectively tend to those of Shannon entropy when 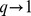.

### Properties of generalized 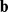 entropy


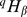
 is not independent of 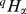. Only Jost's 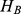 is an independent 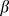 component of diversity indices. But 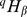 takes place in a generalized decomposition of entropy. Its limit when 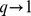 is Shannon 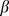 entropy, and in this special case only 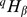 is independent of 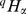.


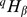
 is interpretable and self-contained (*i.e.* it is not just a function of 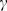 and 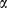 entropies): it is the information gain brought by the knowledge of each local community's species probabilities related to the meta-community's probabilities. It is an entropy, defined just as Shannon 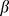 entropy but with a generalized information function.


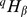
 is always positive (proof in [25]), so entropy decomposition is not limited to equally-weighted communities.

### Bias correction

Estimation bias (we follow the terminology of Dauby and Hardy [26]) is a well-known issue. Real data are almost always samples of larger communities, so some species may have been missed. The induced bias on Simpson entropy is smaller than on Shannon entropy because the former assigns lower weights to rare species, *i.e.* the sampling bias is even more important when 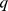 decreases.

We denote 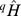 the naive estimators of entropy, obtained by applying the above formulas to estimators of probabilities (such as 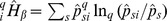). Let 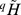 denote the estimation-bias corrected estimators. Chao and Shen's [27] correction can be applied to all of our estimators. It relies on the Horvitz-Thomson [28] estimator which corrects a sum of measurements for missing species by dividing each measurement by 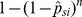, *i.e.* the probability for each species to be present in the sample. Next, the sample coverage of community 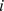, denoted 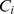, is the sum of probabilities the species of the sample represent in the whole community. It is easily estimated [29] from the number of singletons (species observed once) of the sample, denoted 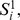 and the sample size 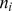:
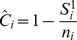
(26)


The sample coverage of the meta-community is estimated the same way: 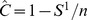. An unbiased estimator of 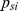 is 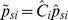, and 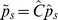. Combining sample coverage, Horvitz-Thomson and equation (23) estimator yields:
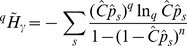
(27)

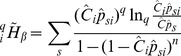
(28)


Another estimation bias has been widely studied by physicists. The latter generally consider that all species of a given community are known and their probabilities quantified. Their main issue is not at all missing species but the non-linearity of entropy measures (see [30] for a short review). Probabilities 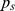 are estimated by 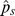. For 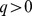, estimating 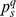 by 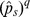 is an important source of underestimation of entropy. Grassberger [31] derived an unbiased estimator 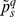 under the assumption that the number of observed individuals of a species along successive samplings follows a Poisson distribution, as in Fisher's model [32] although arguments are different. Grassberger shows that:
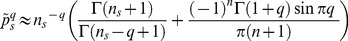
(29)where 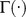 is the gamma function (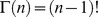 if 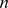 is an integer). Practical computation of 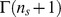 is not possible for large samples so the first term of the sum must be rewritten as: 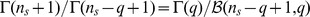 where 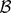 is the beta function. This estimator can be plugged into the formula of Tsallis 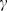 entropy to obtain:
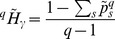
(30)


Other estimations of 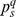 are readily detailed here. Holste *et al.* [33] derived the Bayes estimator of 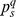 (with a uniform prior distribution of probabilities not adapted to most biological systems) and, recently, Hou *et al.* [34] derived 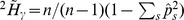, namely the bias correction proposed by Good [29] and Lande [10]. Bonachela *et al.* [30] proposed a balanced estimator for not too small probabilities 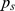 which do not follow a Poisson distribution. This may be applied to low-diversity communities. In summary, the estimation of 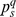 requires assumptions about the distribution of 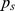 and Grassberger's correction is recognized by all these authors as the best up-to-date for very diverse communities. Better corrections exist but are available for special values of 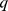 only, such as the recent Chao *et al.*'s estimator of Shannon entropy [35].

The correction for missing species by Chao and Shen and that for non-linearity by Grassberger ignore each other. Chao and Shen's bias correction is important when 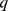 is small and becomes negligible for 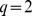 while Grassberger's correction increases with 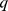, vanishing for 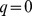. A rough but pragmatic estimation-bias correction is the maximum value of the two corrections. It cannot be applied when 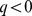 (Grassberger's correction is limited to positive values of 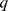) neither to 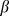 entropy (Chao and Shen's correction can but Grassberger's can't). An estimator of 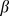 entropy will be obtained as the difference between unbiased 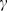 and 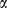 entropy.

**Figure 1:**
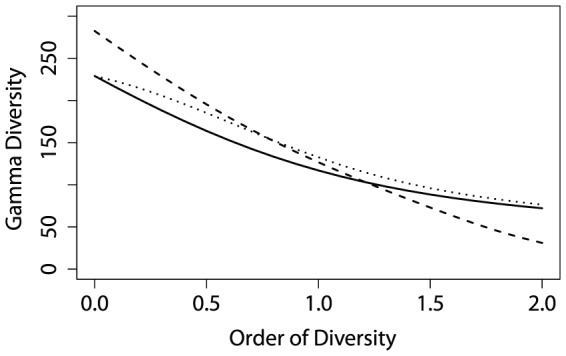
Profile of the 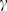 diversity in a tropical forest meta-community. Data from French Guiana, Paracou research station, 2 ha inventoried, 1124 individual trees, and 229 observed species. Solid line: without estimation bias correction; dotted line: Grassberger correction; dashed line: Chao and Shen correction. The maximum value is our bias-corrected estimator of diversity.

We illustrate this method with a tropical forest dataset already investigated by [12]. Two 1-ha plots were fully inventoried in the Paracou field station in French Guiana. This results in 1124 individual trees (diameter at breast height over 10 cm) belonging to 229 species. Figure 1 shows diversity values calculated for 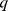 between 0 and 2, with and without correction. Chao and Shen's bias correction is inefficient for 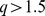 and can even be worse than the naive estimator. In contrast, Grassberger's correction is very good for high values of 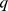, but ignores the missed species and decreases when 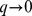. The maximum value offers an efficient correction. By nature, 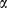 and 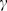 diversity values decrease with 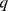 (proof in [36]): around 300 species are estimated in the meta-community (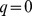, Figure 1), but the equivalent number of species is only 73 for 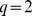.

Converting unbiased entropy into diversity introduces a new bias issue because of the non-linear transformation by the deformed exponential of order 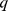. We follow Grassberger's argument: this bias can be neglected because the transformed quantity (i.e. the entropy) is an average value (the information) over many independent terms, so it has little fluctuations (contrarily to the species probabilities whose non-linear transformation causes serious biases, as we have seen above).

**Figure 2:**
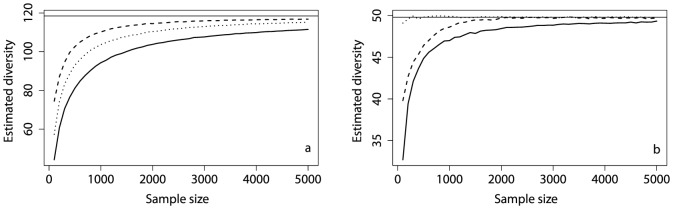
Efficiency of bias correction. Estimation of diversity of the BCI tropical forest plot for two values of the order of diversity 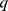 (a: 0.5, b: 1.5). The horizontal line is the actual value calculated from the whole data (around 25000 trees, species frequencies are close to a log-normal distribution). Estimated values are plotted against the sample size (100 to 5000 trees). Solid line: naive estimator with no correction; dotted line: Grassberger correction; dashed line: Chao and Shen's correction. For q = 0.5, Chao and Shen perform best. For q = 1.5, Grassberger's correction is very efficient even with very small samples.

We used Barro Colorado Island (BCI) tropical forest data [37] available in the vegan package [38] for R [39] to show the convergence of the estimators to the real value of diversity. 21457 trees were inventoried in a 50 hectare plot. They belong to 225 species. Only 9 species are observed a single time, so the sample coverage is over 99.99%. The inventory can be considered as almost exhaustive and used to test bias correction. We subsampled the BCI community by drawing chosen size samples (from 100 to 5000 trees) in a multinomial distribution respecting the global species frequencies. We drew 100 samples of each size, calculated their entropy, averaged it and transformed the result into diversity before plotting it in Figure 2. For low values of 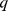, Chao and Shen's correction is the most efficient. It is close to the Chao1 estimator [40] of the number of species for 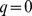 (not shown). A correct estimation of diversity of order 0.5 is obtained with less than 1000 sampled trees (around 2 hectares of inventory). When 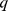 increases, Grassberger bias correction is more efficient: for 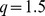 and over, very small samples allow a very good evaluation. Both corrections are equivalent around 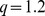 (not shown).

## Examples

### Simple, theoretical example

**Figure 3:**
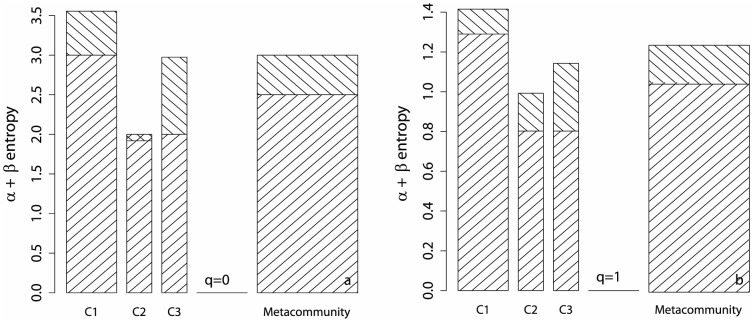
Decomposition of a meta-community entropy. The meta-community is made of three communities named C1, C2 and C3 (described in the text). Their 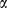 entropy 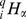 (bottom part of the bars) and their contribution to 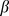 entropy 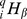 (top part of the bars) are plotted for 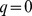 (a) and 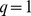 (b). The width of bars is each community's weight. 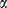 and 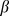 entropies of the meta-community are the weighted sums of those of communities, so the area of the rectangles representing community entropies sum to the area of the meta-community's (width equal to 1). 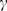 entropy of the meta-community is 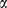 plus 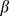 entropy.

We first propose a very simple example to visualize the decomposition of entropy. A meta-community containing 4 species is made of 3 communities C1, C2 and C3 with weights 0.5, 0.25 and 0.25. The number of individuals of each species in communities are respectively (25, 25, 40, 10), (70, 20, 10, 0), (70, 10, 0, 20). The resulting meta-community species frequencies is (0.475, 0.2, 0.225, 0.1). Note that community weights do not follow the number of individuals (100 in each community). No bias correction is necessary since the sample coverage is 1 in all cases. Entropy decomposition is plotted in Figure 3. For 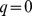, 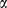 and 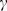 entropy equal the number of species minus 1. The meta-community's 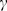 entropy is 3, including 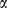 entropy equal to 2.5 (the average number of species minus 1). 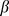 entropy is 0.5, equal to the averaged sum of communities contributions. C2's 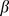 entropy is negative (the total 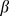 entropy is always positive, but communities contributions can be negative).

Considering Shannon entropy, C1 is still the most diverse community (4 species versus 3 in C2 and C3, and a more equitable distribution: it has the greatest 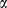 entropy equal to 1.29). C2 and C3 have the same 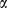 entropy (their frequency distributions are identical) equal to 0.8. C3's species distribution is more different from the meta-community's than the others: it has the greatest 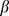 entropy equal to 0.34. Entropies can be transformed into diversities to be interpreted: the 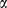 diversity of communities is 3.6, 2.2 and 2.2 effective species, the total 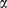 diversity equals 2.8 effective species. The meta-community's 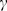 diversity is 3.5 effective species (quite close to its maximum value 4 if all species were equally distributed) and 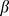 diversity is 1.2 effective communities: the same 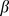 diversity could be obtained with 1.2 theoretical, equally weighted communities with no species in common.

### Real data application

We now want to compare diversity between Paracou and BCI, the two forests introduced in the previous section.

Diversity profiles are a powerful way to represent diversity of communities advocated recently by [36], as a function of the importance given to rare species which decreases with 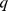. Comparing diversity among communities requires plotting their diversity profiles rather than comparing a single index since profiles may cross (examples from the literature are gathered in [36], Figure 2). Yet, estimation bias depends on the composition of communities, questioning the robustness of comparisons: a consistent bias correction over orders of entropy is required.

**Figure 4:**
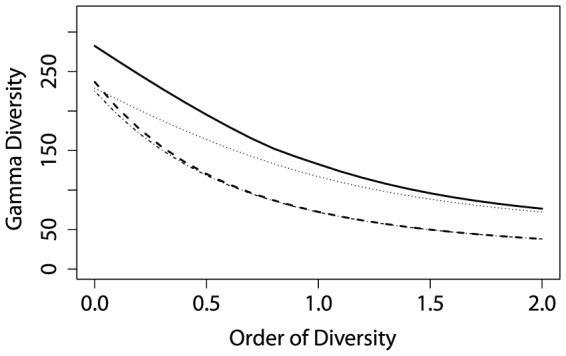
Paracou and BCI 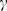 diversity. Diversity of the forest stations is compared. Solid line: Paracou with bias correction; dotted line: Paracou without bias correction; dashed line: BCI with bias correction; dotted dashed line: BCI without bias correction. Without bias correction, Paracou and BCI diversities appear to be similar for low values of 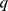. Bias correction shows that Paracou is undersampled compared to BCI (actually around 1000 trees versus 25000). Paracou is much more diverse than BCI.


**Figure 5:**
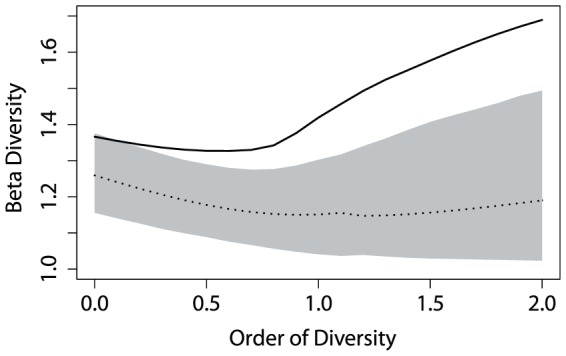
Paracou and BCI 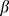 diversity. 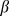
 diversity profile between Paracou plots (solid line) is compared to that of any two plots of BCI (dotted line with 95% confidence envelope).

Entropy is converted to diversity and plotted against 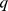 in Figure 4 for our two forests: plots are given equal weight since they have the same size and gamma diversity is calculated for each meta-community. Paracou is more diverse, whatever the order of diversity. Bias correction allows comparing very unequally sampled forests (2 ha in Paracou versus 50 ha in BCI, sample coverage equal to 92% versus 99.99%).

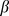
 diversity profile is calculated between the two plots of Paracou. To compare it with BCI which contains 50 1-ha plots, we calculated 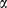 and 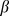 entropies between all couples of BCI plots, averaged them and converted them into 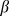 diversity (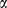 and 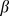 entropies are required to calculate 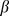 diversity). We also calculated the 95% confidence envelope of 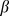 diversity between two 1-ha plots of BCI by eliminating the upper and lower 2.5% of the distribution of all plot couples 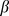 diversity. We chose to use Chao and Shen's correction up to 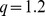 and Grassberger's correction for greater 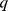 to obtain comparable results in the 1225 pairs of BCI plots. Figure 5 shows Paracou's 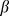 diversity is greater than BCI's, especially when rare species are given less importance: for 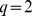 (Simpson diversity), two plots in BCI are as different from each other as 1.2 plots with no species in common, while Paracou's equivalent number of plots is 1.7. In other words, dominant species are very different in Paracou plots, while they are quite similar on average between two BCI plots.

The shape of 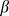 diversity profiles is more complex than that of 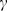 diversity. At 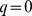, 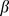 diversity equals the ratio between the total number of species and the average number of species in each community [7]. At 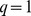, it is the exponential of the average Kullback-Leibler divergence between communities and the meta-community. A minimum is reached between both. Over 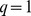, 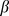 diversity increases to asymptotically reach its maximum value equal to 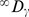, *i.e.* the inverse of the probability of the most frequent species of the meta-community, divided by 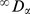, *i.e.* the inverse of the probability of the most frequent species in each community.

## Discussion

Diversity can be decomposed in several ways, multiplicatively, additively or non-additively if we focus on entropy. A well-known additive decomposition of Simpson entropy is as a variance (that of Nei [41] among others). It is derived in Appendix S2. It is not a particular case of our generalization: the total variance between communities actually equals 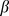 entropy but the relative contribution of each community is different. Among these several decompositions, only the multiplicative partitioning of equally-weighted communities (17) and the non-additive partitioning of entropy (19) allow independent 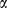 and 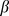 components (except for the special case of 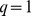), but unequal weights are often necessary and ecologists may not want to restrict their studies to Shannon diversity.

We clarify here the differences between non-additive partitioning and our additive decomposition and we address the question of unequally-weighted communities.

### Additive versus non-additive decomposition

Jost [3] focused on independence of the 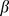 component of the partitioning. He showed (appendix 1 of [3]) that if communities are not equally weighted the only definition of 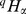 allowing independence between 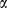 and 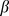 components is 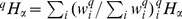. The drawback of this definition is that 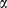 may be greater than 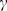 entropy if 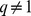 and community weights are not equal. Each component of entropy partitioning can be transformed into diversity as a Hill number.

We have another point of view. We rely on Patil and Taillie's concept of diversity of a mixture (section 8.3 of [14]), which implies Routledge's definition of 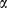 entropy. It does not allow independence between 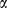 and 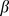 components of the decomposition except for the special case of Shannon entropy, but it ensures that 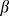 entropy is always positive. We believe that independence is not essential when dealing with entropy, as it emerges when converting entropy to diversity, at least when community weights are equal. The 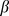 component of the decomposition cannot be transformed into 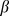 diversity without the knowledge of 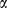 entropy but we have shown that it is an entropy, justifying the additive decomposition of Tsallis entropy.

The value of 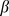 entropy cannot be interpreted or compared between meta-communities as shown by [4], but combining 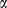 and 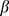 entropy allows calculating 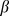 diversity (Table 1).

### Unequally weighted communities

Routledge's definition of 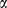 entropy does not allow independence between 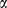 and 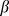 diversity when community weights are not equal, and 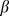 diversity can exceed the number of communities [7]. We show here that the number of communities must be reconsidered to solve the second issue. We consider the independence question then.

We argue that Routledge's definition always allows to reduce the decomposition to the equal-weight case. Consider the example of Chao *et al.* [7]: two communities are weighted 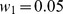 and 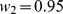, their respective number of species are 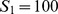 and 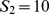, no species are shared, and we focus on 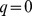 for simplicity. 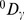 equal 110 species, 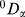 is the weighted average of 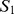 and 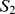 equal to 14.5, so 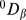 is 7.6 effective communities, which is more than the actual 2 communities. But this example is equivalent to that of a meta-community made of 1 community identical to the first one and 19 communities identical to the second one, all equally weighted. 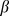 diversity of this 20-community meta-community is 7.6 effective communities.

A more general presentation is as follows. A community of weight 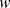 can be replaced by any set of 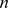 identical communities of weights 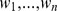 provided that the sum of these weights is 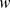, without changing 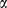, 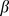 and 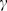 diversity of the meta-community because of the linearity of Routledge's definition of entropy. Any unequally weighted set of community can thus be transformed into an equally weighted one by a simple transformation (strictly speaking, if weights are rational numbers).

Consider a meta-community made of several communities with no species in common, and say the smallest one (its weight is 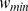) is the richest (its number if species is 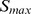). If 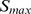 is large enough, the number of species of the meta-community is not much more than it (poor communities can be neglected). 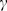 richness 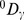 tends to 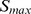, 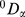 tends to 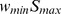, so 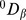 tends to 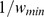. The maximum value 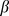 diversity can reach is the inverse of the weight of the smallest community: its contribution to 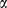 diversity is proportional to its weight, but its contribution to 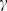 diversity is its richness. Given the weights, the maximum value of 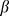 diversity is thus 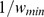; it is the number of communities if weights are equal.

Comparing 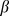 diversity between meta-communities made of different number of communities is not possible without normalization. Jost [3] suggests normalizing it to the unit interval by dividing it by the number of communities in the equal-weight case. We suggest extending this solution to dividing 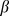 diversity by 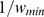. When weights are not equal, the number of communities is not the appropriate reference.

Although we could come back to the equally-weighted-community partition case, 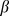 diversity is not independent of 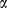 diversity because communities are not independent of each other (some are repeated). Chao *et al.* (appendix B1 of [7]) derive the relation between the maximum value of 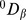 and 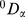 for a two-community meta-community: 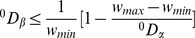. The last term quantifies the relation between 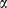 and 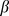 diversity. It vanishes when weights are close to each other, and it decreases quickly with 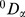. If 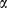 diversity is not too low (say 50 species), the constraint is negligible (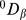 can be greater than 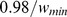 whatever the weights).

A complete study of the dependence between 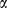 and 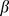 diversity for all 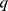 values and more than two communities is beyond the scope of this paper but these first results show that this dependence is not so serious a problem as that between 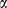 and 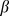 entropy. As long as weights are not too unequal and diversity is not too small, results can be interpreted clearly.

Very unequal weights imply lower 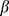 diversity: the extreme case is when the larger community is the richest. If it is large enough, the meta-community is essentially made of the largest community and 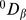 tends to 1. This is not an issue of the measure, but a consequence of the sampling design.

## Conclusion

The additive framework we proposed here has the advantage of generalizing the widely-accepted decomposition of Shannon entropy, providing a self-contained definition of 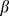 entropy and some ways to correct for estimation biases. Deformed logarithms allow a formal parallelism between HCDT and Shannon entropy (equations (15) and (16) and Table 1). Of course, diversity can be calculated directly, but no estimation-bias correction is available then. The additive decomposition of HCDT entropy can be considered empirically as a calculation tool whose results must systematically be converted to diversity for interpretation.

We rely on Routledge's definition of 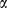 entropy which allows decomposing unequally-weighted communities and takes place in a well-established theoretical framework following Patil and Taillie. The price to pay is some dependence between 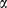 and 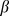 diversity when weights are not equal. It appears to be acceptable since it is unlikely to lead to erroneous conclusions. Still, a rigorous quantifying of it shall be the object of future research.

We only considered communities where individuals were identified and counted, such as forest inventories. Entropy decomposition remains valid when frequencies only are available but our bias correction relies entirely on the number of individual: other techniques will have to be developed for these communities if unobserved species cannot be neglected. Bias correction is still an open question. We proposed a first and rough solution. More research is needed to combine the available approaches rather than using each of them in turn.

We provide the necessary code for R to compute the analyses presented in this paper as a supplementary material in Appendix S4 with a short user's guide in Appendix S3.

## Supporting Information

Appendix S1 **Detailed derivation of the partitioning.**
(PDF)

Appendix S2 **Decomposition of Simpson index.**
(PDF)

Appendix S3 **Using the code: short user's guide.**
(PDF)

Appendix S4 **R code to compute the analyses.**
(ZIP)
