# Supplementary material for: Generalization of the Partitioning of Shannon Diversity
Source: PLoS One. 2014 Mar 6;9(3):e90289. doi: 10.1371/journal.pone.0090289 (PMC3946064; doi:10.1371/journal.pone.0090289)
Supplement: Appendix S1 — Detailed derivation of the partitioning. (PDF) [file pone.0090289.s001.pdf]

Appendix S1: Detailed derivation of the partitioning

We present here the fully detailed derivation of the partitioning of entropy, summarized in the Methods section. We start from the multiplicative decomposition of true diversity:

$${}^{q}D_{\gamma} = {}^{q}D_{\alpha}\,{}^{q}D_{\beta}$$

$$\Leftrightarrow \ln_q {}^{q}D_{\gamma} = \ln_q {}^{q}D_{\alpha} + \ln_q {}^{q}D_{\beta} - (q-1)\left(\ln_q {}^{q}D_{\alpha}\right)\left(\ln_q {}^{q}D_{\beta}\right)$$

We factorize the last two terms.

$$\Leftrightarrow {}^{q}H_{\gamma} = \ln_q {}^{q}D_{\alpha} + \left(\ln_q {}^{q}D_{\beta}\right)\left[1 - (q-1)\ln_q {}^{q}D_{\alpha}\right]$$

We replace $\ln_q {}^{q}D_{\alpha}$ by its value (equations (7) and (9)):

$$\Leftrightarrow {}^{q}H_{\gamma} = \frac{1 - \sum_i w_i \sum_s p_{si}^{q}}{q-1} + \left(\ln_q {}^{q}D_{\beta}\right)\left[1 - (q-1)\frac{1 - \sum_i w_i \sum_s p_{si}^{q}}{q-1}\right]$$

We can factorize the first term because $\sum_i w_i = 1$ and simplify the second term:

$$\Leftrightarrow {}^{q}H_{\gamma} = \sum_i w_i \frac{1 - \sum_s p_{si}^{q}}{q-1} + \left(\ln_q {}^{q}D_{\beta}\right)\left(\sum_i w_i \sum_s p_{si}^{q}\right)$$

We replace $\ln_q {}^{q}D_{\beta}$ by $\ln_q\left({}^{q}D_{\gamma}/\,{}^{q}D_{\alpha}\right)$ and introduce probabilities following equations (6) and (7):

$$\Leftrightarrow {}^{q}H_{\gamma} = \sum_i w_i \frac{1 - \sum_s p_{si}^{q}}{q-1} + \frac{1 - \frac{\sum_s p_s^{q}}{\sum_i w_i \sum_s p_{si}^{q}}}{q-1}\left(\sum_i w_i \sum_s p_{si}^{q}\right)$$

We recognize ${}_{i}^{q}H_{\alpha}$ in the first term and the last term simplifies:

$$\Leftrightarrow {}^{q}H_{\gamma} = \sum_i w_i\, {}_{i}^{q}H_{\alpha} + \frac{\sum_i w_i \sum_s p_{si}^{q} - \sum_s p_s^{q}}{q-1}$$

The last step consists in reducing the power of p_s^{q} by 1, since $\sum_s p_s^{q} = \sum_s p_s^{q-1} \sum_i w_i\, p_{si}$:

$$\Leftrightarrow {}^{q}H_{\gamma} = \sum_i w_i\, {}_{i}^{q}H_{\alpha} + \sum_i w_i \frac{\sum_s\left(p_{si}^{q} - p_s^{q-1} p_{si}\right)}{q-1} = \sum_i w_i\, {}_{i}^{q}H_{\alpha} + \sum_i w_i \frac{\sum_s p_{si}^{q}\left[1 - \left(\frac{p_{si}}{p_s}\right)^{1-q}\right]}{q-1}$$

$$\Leftrightarrow {}^{q}H_{\gamma} = \sum_i w_i\, {}_{i}^{q}H_{\alpha} + \sum_i w_i \sum_s p_{si}^{q} \ln_q \frac{p_{si}}{p_s}$$

Before the last step, calculation id identical with Jost's weighting of α diversity, replacing w_i by w_i^{q} everywhere, but Routledge's weighting is necessary for the second term to be a weighted sum of community generalized Kullback-Leibler divergences that we will identify to ${}^{q}H_{\beta}$ (1, in red on the penultimate line, would be w_i^{q-1}).
