# Supplementary material for: Generalization of the Partitioning of Shannon Diversity
Source: PLoS One. 2014 Mar 6;9(3):e90289. doi: 10.1371/journal.pone.0090289 (PMC3946064; doi:10.1371/journal.pone.0090289)
Supplement: Appendix S2 — Decomposition of Simpson index. (PDF) [file pone.0090289.s002.pdf]

Appendix S2: Decomposition of Simpson index

We use Pélissier and Couteron's [1] organization of data to derive the decomposition. We define an indicator $\mathbf{1}_{sk}$ such that it takes a value of 1 if individual k belongs to species s, and of zero otherwise. $\mathbf{1}_{sk}$ follows a Bernoulli distribution: either an individual belongs to species s or not. $\sum_{k=1}^{n} \mathbf{1}_{sk}$ follows a binomial distribution of variance $np_s(1-p_s)$. A standard partitioning of variance can be applied. Within each community, variance is $n_i p_{si}(1-p_{si})$. The expectation of $\sum_{k=1}^{n} \mathbf{1}_{sk}$ is np_s for pooled data, and that of within-community $\sum_{k=1}^{n_i} \mathbf{1}_{sk}$ is $n_i p_{si}$. We have then:

$$p_s(1-p_s) = \sum_i \frac{n_i}{n} \left[p_{si}(1-p_{si}) + (p_{si}-p_s)^2 \right] \tag{1}$$

Summation of $p_s(1-p_s)$ over species provides Simpson's entropy:

$$^2H_\gamma = \sum_i \frac{n_i}{n} {}_i^2H_\alpha + \sum_i \frac{n_i}{n} \sum_s (p_{si}-p_s)^2 \tag{2}$$

Assuming $^2H_\gamma = {}^2H_\alpha + {}^2H_\beta$ and α entropy is the weighted sum of within-community α entropy values, the second term of (2) can be identified to β entropy in the classical additive partitioning. $^2H_\beta$ is the weighted sum of contributions of communities, denoted ${}_i^2H_\beta = \sum_s (p_{si}-p_s)^2$. This additive partitioning of entropy is that of Nei [2] among others. As shown by Jost [3,4], $^2H_\beta$is constrained by $^2H_\alpha$ since γ entropy is limited to 1. Using $^2H_\beta$ or $G_{ST} = {}^2H_\beta / {}^2H_\gamma$ as a measure of differentiation has been shown to be erroneous [5-7].

References:

1. Pélissier R, Couteron P (2007) An operational, additive framework for species diversity partitioning and beta-diversity analysis. Journal of Ecology 95: 294-300.
2. Nei M (1973) Analysis of Gene Diversity in Subdivided Populations. Proceedings of the National Academy of Sciences of the United States of America 70: 3321-3323.
3. Jost L (2006) Entropy and diversity. Oikos 113: 363-375.
4. Jost L (2007) Partitioning diversity into independent alpha and beta components. Ecology 88: 2427-2439.
5. Jost L (2008) G_{ST} and its relatives do not measure differentiation. Molecular Ecology 17: 4015-4026.
6. Jost L (2009) D vs. G_{ST}: Response to Heller and Siegismund (2009) and Ryman and Leimar (2009). Molecular Ecology 18: 2088-2091.
7. Heller R, Siegismund HR (2009) Relationship between three measures of genetic differentiation G(ST), D-EST and G'(ST): how wrong have we been? Molecular Ecology 18: 2080-2083.
