# Supplementary material for: Generalization of the Partitioning of Shannon Diversity
Source: PLoS One. 2014 Mar 6;9(3):e90289. doi: 10.1371/journal.pone.0090289 (PMC3946064; doi:10.1371/journal.pone.0090289)
Supplement: Appendix S3 — Using the code: short user's guide. (PDF) [file pone.0090289.s003.pdf]

Appendix S3: Using the code

The R code provided in Appendix S4 is fully commented. Data should be imported from two text (csv) files containing the species abundances and the community weights. If the dataset of Appendix S4 is unzipped in R working directory, all examples and figures of the paper can be run.

The main four functions are:

- *MetaCommunity*: to transform abundances and weights into an object containing all summary statistics necessary to calculate diversity.
- *DivPart*: to partition the diversity of a metacommunity (Figure S1).

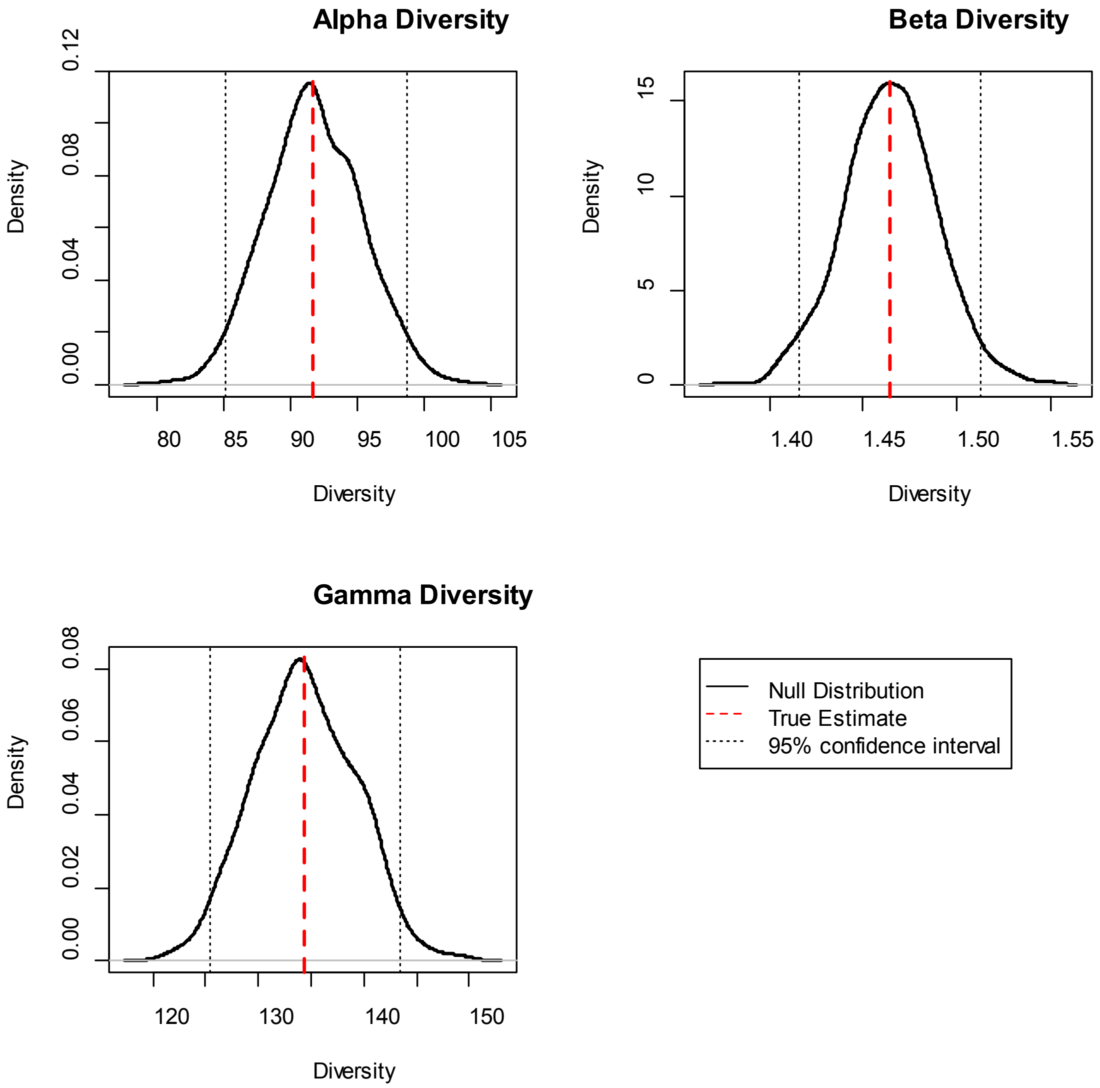

Figure S1: output of *DivPart* for the example metacommunity.

- *DivEst*: to partition and estimate confidence intervals of estimators by redistributing abundances according to a multinomial distribution respecting.
- *DivProfile*: to draw diversity profiles (Figure S2).

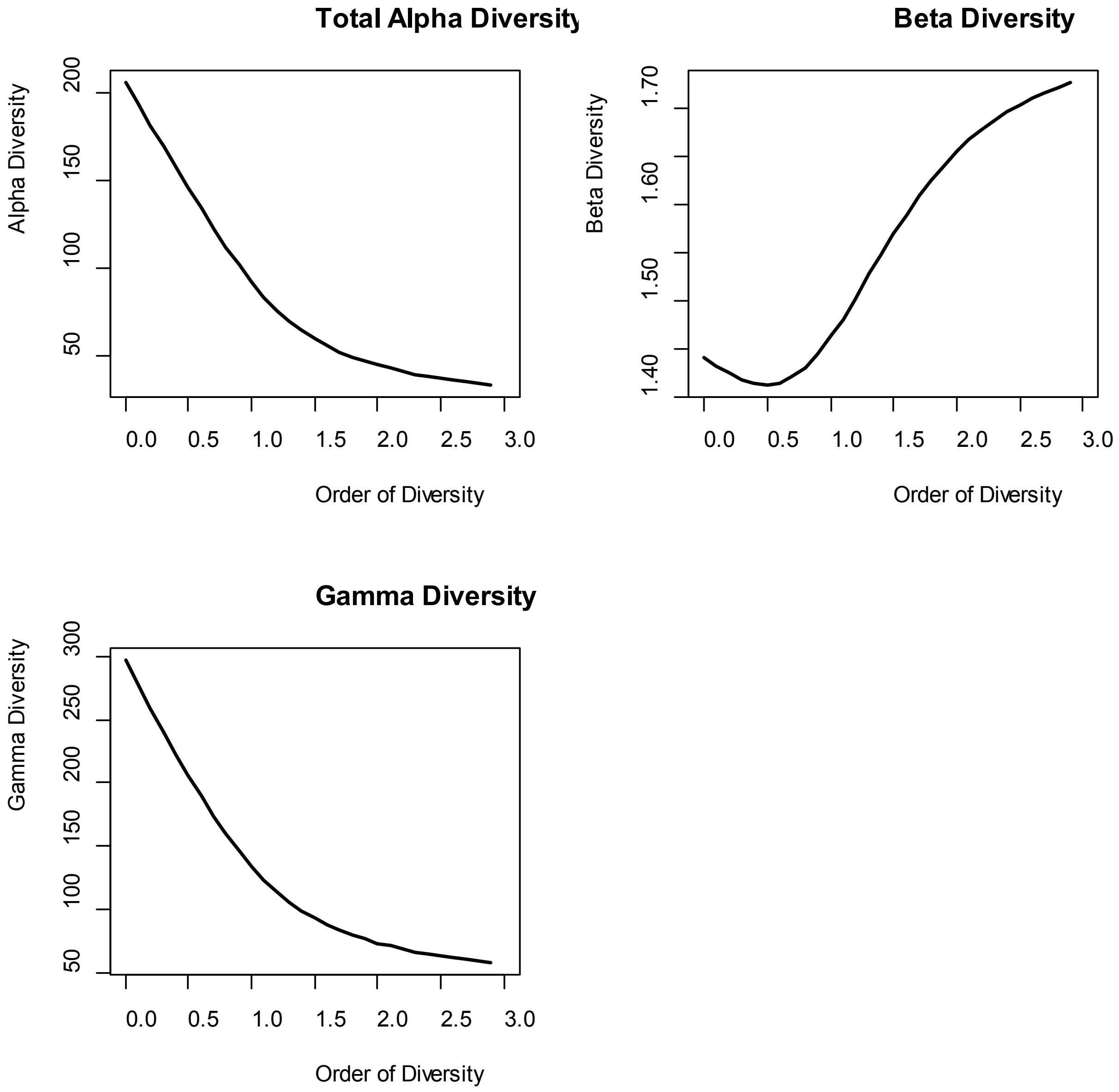

Figure S2: output of *DivProfile* for the example metacommunity.

A special attention must be paid to the bias-correction choice. "*Best*" is the default value: it applies the correction described in the paper for α and γ diversity, and calculates β entropy (diversity) as the difference (ratio) between γ and α since the correction for β entropy is currently limited to Chao and Shen's.
